# Sulfur-isotope anomalies recorded in Antarctic ice cores as a potential proxy for tracing past ozone layer depletion events

**DOI:** 10.1093/pnasnexus/pgac170

**Published:** 2022-08-30

**Authors:** Sanjeev Dasari, Guillaume Paris, Julien Charreau, Joel Savarino

**Affiliations:** Institut des Géosciences de l'Environnement (IGE), University Grenoble Alpes, CNRS, IRD, Grenoble INP, Grenoble 38000, France; Centre de Recherches Pétrographiques et Géochimiques, Université de Lorraine, CNRS, 54000 Nancy, France; Centre de Recherches Pétrographiques et Géochimiques, Université de Lorraine, CNRS, 54000 Nancy, France; Institut des Géosciences de l'Environnement (IGE), University Grenoble Alpes, CNRS, IRD, Grenoble INP, Grenoble 38000, France

**Keywords:** cosmic-ray background, UV radiation, sulfur mass-independent fractionation (S-MIF), Δ^33^S

## Abstract

Changes in the cosmic-ray background of the Earth can impact the ozone layer. High-energy cosmic events [e.g. supernova (SN)] or rapid changes in the Earth's magnetic field [e.g. geomagnetic Excursion (GE)] can lead to a cascade of cosmic rays. Ensuing chemical reactions can then cause thinning/destruction of the ozone layer—leading to enhanced penetration of harmful ultraviolet (UV) radiation toward the Earth's surface. However, observational evidence for such UV “windows” is still lacking. Here, we conduct a pilot study and investigate this notion during two well-known events: the multiple SN event (≈10 kBP) and the Laschamp GE event (≈41 kBP). We hypothesize that ice-core-Δ^33^S records—originally used as volcanic fingerprints—can reveal UV-induced background-tropospheric-photochemical imprints during such events. Indeed, we find nonvolcanic S-isotopic anomalies (Δ^33^S ≠ 0‰) in background Antarctic ice-core sulfate during GE/SN periods, thereby confirming our hypothesis. This suggests that ice-core-Δ^33^S records can serve as a proxy for past ozone-layer-depletion events.

Significance Statement The ozone layer in the upper troposphere–lower stratosphere (UTLS) region absorbs ultraviolet (UV) radiation, thereby limiting the penetration of harmful UV rays into the Earth's atmosphere. However, in the event of destruction or thinning of the ozone layer, this harmful UV radiation could penetrate deeper into the troposphere, causing detrimental effects on humans. Here, we provide observational evidence of changes in the cosmic-ray background of the Earth during past events such as magnetic field collapses and Supernovas, having affected the ozone layer. As such, future events of a similar kind could potentially lead to UV-induced environmental effects.

## Introduction

The cosmic-ray background of the Earth is modulated mainly by solar activity, galactic cosmic rays, and the geomagnetic field (1). Radionuclides, such as carbon-14 (^14^C), beryllium-10 (^10^Be), and chlorine-36 (^36^Cl), are powerful proxies often used to investigate changes in the cosmic-ray background (2, 3). Changes to this background usually occur on decadal to millennial scales (e.g. from solar minima/maxima and variations in the incoming flux of galactic cosmic rays) (4). Certain high-energy solar events producing a cascade of cosmic rays have also led to a rapid, short-term increase in the atmospheric production of radionuclides as seen during, e.g. ≈ 660 BC, 774/775 AD, and 993/994 AD from environmental archives such as tree rings and ice cores (5–9). Solar proton event(s), e.g. (5–9) are associated with these rapid increases in such cosmic-ray tracers. Recently, an apparent supernova (SN) causation has also been suggested (10). SNs are an immediate release of ≈10^51^ ergs of energy, the result of either the catastrophic collapse of a massive star or runaway nuclear burning on the surface of a white dwarf (10, 11). They are prompt sources of atomic nuclei accelerated to high energies, including extreme ultraviolet (UV) and gamma rays. Depending on the distance, SNs (and the subsequently formed remnants, SNRs) could produce a cosmic-ray flux of sufficient intensity to affect the radioisotope signatures on Earth (10) and thus pose an existential threat to life on Earth's surface (11). In contrast, the Earth's magnetic field deflects much of the cosmic radiation reaching the Earth, thereby acting as a shield for life on the planet (12). However, variations in the Earth's magnetic dipole moment can affect this shielding capacity and lead to prolonged changes in the cosmic-ray background. On geological timescales, the Earth's magnetic dipole moment has varied greatly in direction, polarity, and intensity (13). Particularly rapid events during which Earth's magnetic dipole moment, and thus its shielding capacity, collapsed are known. These events can be associated with geomagnetic excursions (GEs) such as ≈41 ky ago (Laschamp) and ≈190 ky ago (Iceland-Basin) (14). GEs can last from a few hundred to a few thousand years and remain one of the less well-understood aspects of magnetic field behavior (14).

The effect of variations in the cosmic-ray background on the ozone layer remains sparsely investigated and poorly understood (15). The ozone layer, located in the upper troposphere lower stratosphere region, absorbs UV radiation, thereby limiting the penetration of UV rays into Earth's atmosphere (16). It thus reduces exposure to this UV radiation, which would otherwise increase the risk of carcinogenesis (cataract and skin cancer), cause immune system suppression, and general DNA damage in humans (16). However, in the event of destruction or thinning of the ozone layer, this harmful UV radiation could penetrate deeper into the troposphere, causing detrimental effects on humans. This possibility exists during high-energy cosmic events (e.g. SNs) or the magnetic field collapses (e.g. GEs). These events could lead to an increase in the cosmic-ray bombardment, and through enhanced chemical effects and the production of nitric oxide (NO) in the upper atmosphere (≈50 to 120 km), causing a thinning and even destruction of the ozone layer (15, 16).

A collapse of the Earth's shielding capacity, seen in the past (13), could also occur in the foreseeable future (17). Both natural causes, such as the decreasing intensity of Earth's magnetic field (18, 19), and human causes, such as disruption to portions of the geomagnetic field by nuclear detonations or the ionosphere through focused electromagnetic radiation, have been proposed (17). It thus remains crucial to investigate if this anticipated drop in the shielding capacity of the Earth would be (or would not be) associated with an effect on the ozone layer. Likewise, based on theoretical and modeling calculations, SNs have also been advocated as potential candidates for ozone depletion (11, 20, 21). To better assess future GE-/SN-based ozone depletion scenarios, knowledge of past events, which could aid in envisaging both the likelihood and prevalence of such an effect, is imperative.

The existence of thinning/destruction of the ozone layer in connection with a past GE or SN event has never been confirmed by natural observations despite their potential implications on the environment, ecology, and globally life on Earth (14, 16, 22). It has only been inferred from modeling scenarios (20, 21, 23, 24) and climate proxies (25, 26). Physio-chemical models of the atmosphere estimate change in the ozone concentration, as low as 5% to as high as 30%, during changes in the Earth's shielding capacity (21, 23, 24). Paleoclimate proxies have documented events of temporary ozone loss with high levels of UV penetration in Earth's history (359 Ma ago) (25). The evolution of cyanobacterium pigments measured in a lake in Antarctica also suggest high UV exposure during the last glacial stage compared to the Holocene (26). However, the lake sediments studied are much younger than some major GE and SN events and remain poorly dated, offering insufficient temporal resolution.

Here, using natural observations, we aim to investigate the impact on the ozone layer during two past GE and SN events. The globality of an event remains an important qualifier. The Laschamp GE event makes a strong case for this pilot investigation. This sensu stricto geomagnetic event was global and synchronous since it has been observed in many lavas of similar ages in New Zealand and California (14, 27). It has been highlighted in marine stratigraphic records (13) and ice-core records from Greenland and Antarctica (28–31) by tracing the radionuclide ^10^Be. The Laschamp GE represents one of the most important collapses in magnetic dipole moment known since ≈−780 ky and the Brunhes–Matuyama inversion (13, 14, 27). During this period, the intensity of the Earth's magnetic field decreased by more than 80%, going from ≈12 to <1 × 10^22^ A /m^2^ (13). The period ≈41 ky was also associated with the highest recorded changes in the Δ^14^C record (24, 32). Before ≈41 ky ago, a steep rise in cosmogenic radionuclides could be seen during several short periods (e.g. in the IntCal20 results for Δ^14^C) (32). Of particular interest are Δ^14^C anomalies consistent with the SN causation. One such period was ≈10 ky ago when a steep and sustained increase in both Δ^14^C and authigenic ^10^Be/^9^Be was seen over 200 y (10, 13, 32). The Boomerang SN coincides in age and is close enough (≈0.80 kpc) to have plausibly generated enough Earth-incident ψ emission to have caused such anomalies in the cosmogenic nuclides (10). The Boomerang SN event was synchronous with another SN event (based on SN remnant G89.0 + 4.7) of appropriate age but at a greater distance (≈1.25 ± 0.45 kpc) (10). These events are concomitant also with a double-hump pattern in the Δ^14^C record (10, 32). Taken together, the period ≈10 ky ago coincides with multiple SN event. It presents a compelling case study for SN causation of changes in the cosmic-ray background and thereby for plausible thinning/destruction of the ozone layer.

**Fig. 1.**
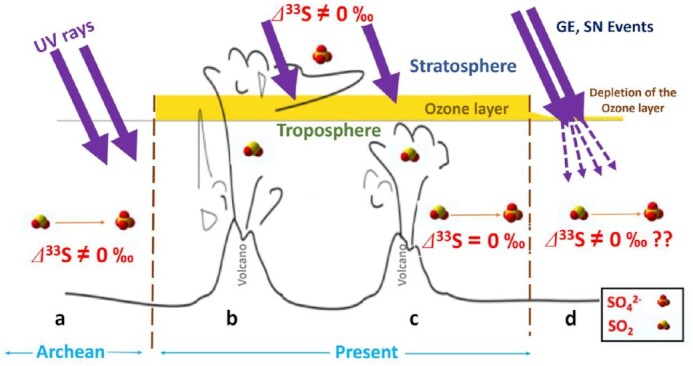
Conceptual illustration of hypothesis for S-isotope-based fingerprinting the depletion of the ozone layer: Sulfur has four stable isotopes: ^32^S, ^33^S, ^34^S, and ^36^S. The isotopic composition of sulfur in a sample is most often reported in the form δ^*x*^S, where *x* can be 33, 34, or 36: δ^*x*^S = 1/[(*^x^*S/^32^S)_sample_/(^*x*^S/^32^S)_reference_] (36). Most of the isotopic fractionation mechanisms for sulfur fractionate isotopes are in proportion to their mass, according to the so-called “mass dependent” fractionation laws (MDF) (36–39). For MDF, one can write δ^33^S ≈ 0.515 × δ^34^S (for its linear approximation), and the relation can be quantified by the value *Δ*^33^S = δ^33^S − [(δ^34^S + 1)^0.515^ − 1] (39). If *Δ*^33^S≠0, one speaks of “mass-independent” fractionation (MIF) (39). On Earth, sulfur isotope composition in most of the rocks and environmental samples is characterized by MDF, with the notable exception of rocks older than ≈2.3 Ga (Archean), in which we find values of *Δ*^33^S between −5‰ and +12‰ (39). Such values then disappear from the sediment records. These values reflect the absence of oxygen in the atmosphere, resulting from the absence of ozone (a) (39). In the absence of ozone, the UV wavelengths between 190 and 250 nm are no longer absorbed by ozone but can be absorbed by sulfur dioxide (SO_2_) or carbonyl disulfide (CS_2_) (38). The ensuing photochemical processes (photolysis or photo-oxidation depending on the exact wavelengths involved) lead to the synthesis of S-MIF-bearing sulfate aerosols (37–39). It is important to note that environmental factors or climate cannot lead to S-MIF splits. In the present atmosphere, the presence of the ozone layer prevents the creation of such large-scale fractionation (b and c). However, a notable exception exists wherein sulfur compounds can be exposed to UV radiation. During volcanic explosions, the emitted SO_2_ starts with a mass-dependent composition (*Δ*^33^S = 0‰). It acquires a mass-independent composition (*Δ*^33^S ≠ 0‰) if and when subjected to photo-oxidation/photolysis (conversion of SO_2_ to sulfate) by shortwave UV radiation that is present only in and above stratospheric ozone layer (41–46) (b), but carries a mass-dependent composition if oxidized below this ozone layer (whose concentration is maximum at 16 to 25 km from polar to tropical regions, respectively) (45) (c). Part of the aerosols thus formed, carrying *Δ*^33^S values other than zero, are then found and analyzed in ice cores or snow deposits in, e.g. Antarctica (41–46). Therefore, this independent isotopic approach allows for identifying stratospheric (b) vs. tropospheric (c) origins of sulfate aerosols (41–46). This approach has been used to fingerprint the history of volcanic eruptions on Earth's surface (41–46). (d) The hypothesis that during the GE and SN events, a strong thinning/destruction of the ozone layer would lead to the creation of a UV “window” leading to deeper penetration of UV radiation toward the Earth's surface (12, 14–16). The UV-induced photochemistry can then generate measurable S-MIF splits in tropospheric sulfate produced from S-containing compounds such as SO_2_, CS_2_, which can be detected in polar ice-core sulfate records for the corresponding periods. For the hypothesis to be valid, the S-MIF should occur in “nonvolcanic” periods in ice-core sulfate.

We explore this putative possibility along with the Laschamp GE event. We hypothesize that a geochemical tracer can be used as a potential proxy for establishing a link between the process of GE and SN events leading to the thinning/destruction of the ozone layer and the enhanced incidence of UV radiation in the troposphere (Figure 1). While some tracers, ice-core nitrate, have been proposed, the choice has been rejected as insufficient to document the extended record of the frequency, energy distribution, and fluence of cosmic events (33–35). This study tests a novel and more direct approach of using observationally constrained stable sulfur isotopic signatures (36–39) (Figure 1a to d). Based on the wealth of literature on the sulfur-mass independent fractionation (S-MIF) in ice-core/aerosol science (37–53) (discussed below, also see Figure 1a to c), it is logical to envisage that a sizeable thinning/destruction of the ozone layer will likely generate a measurable S-MIF anomaly (Δ^33^S) in tropospheric sulfate aerosols due to the penetration of UV rays toward Earth's surface (as depicted in Figure 1d). We can detect these anomalies by analyzing the corresponding period's triple S-isotopic composition (^32^S,^33^S, ^34^S) of aerosol sulfate trapped in ice cores. The ice-core sulfate isotope records have been used as an unambiguous tracer to fingerprint past volcanic eruptions (41–46). The presence (absence) of such an anomaly during periods of “nonvolcanic” influence (as in Figure 1d) would confirm (negate) our hypothesis. Therefore, this pilot investigation explores the possibility of using the S-isotope ice-core records for a new purpose altogether. Our findings might improve understanding of the interactions between cosmic events and the ozone layer and, thereby, the associated environmental stress—a link that remains less explored.

## Results

**Fig. 2.**
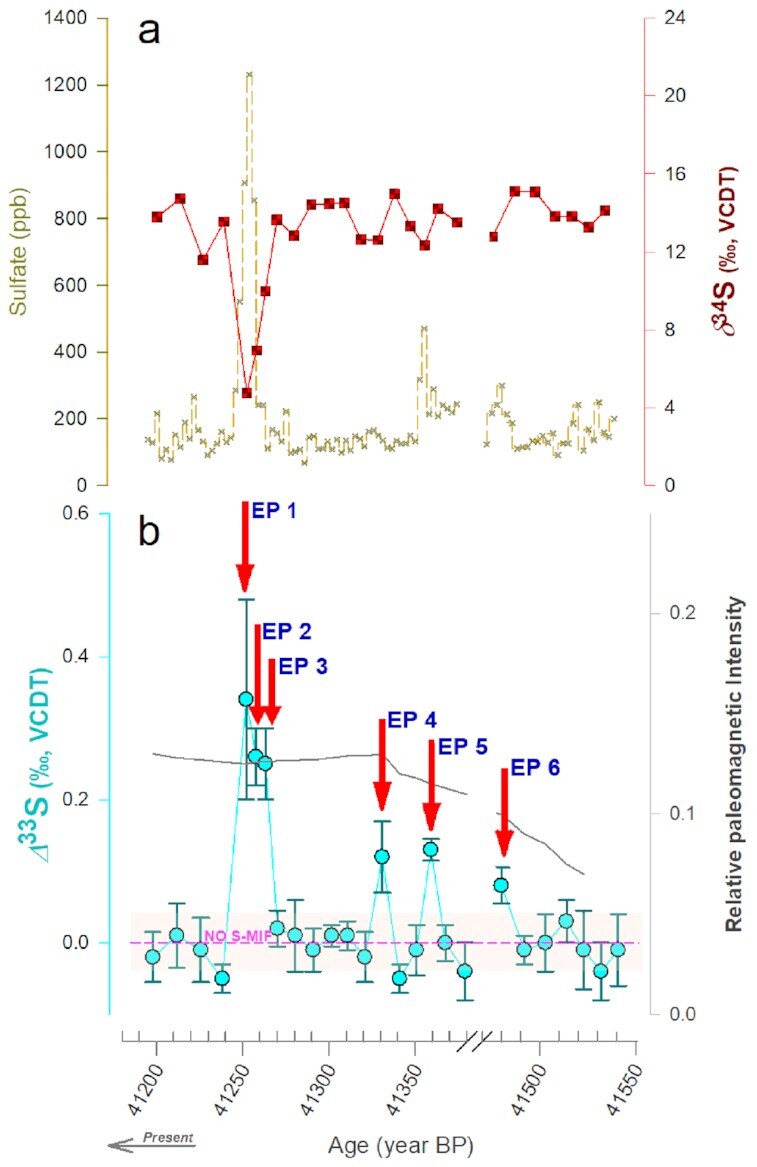
The evolution of ice-core sulfate isotopic signals during the Laschamp GE event. (a) Sulfate concentrations and δ^34^S values are reported (see more details in Table S1) (b) *Δ*^33^S values are reported along with relative paleomagnetic intensity (gray line) (24). The episodes marked with red arrows (“EPs”) highlight the case studies detailed in the discussion. The non-sea-salt sulfate source fractions are shown in Figures S1 and S2. The sampling of the volcanic event (41,240 to 41,270 BP) is shown in greater detail in Figure S3. The AICC2012 chronology has been used (70, 71).

**Fig. 3.**
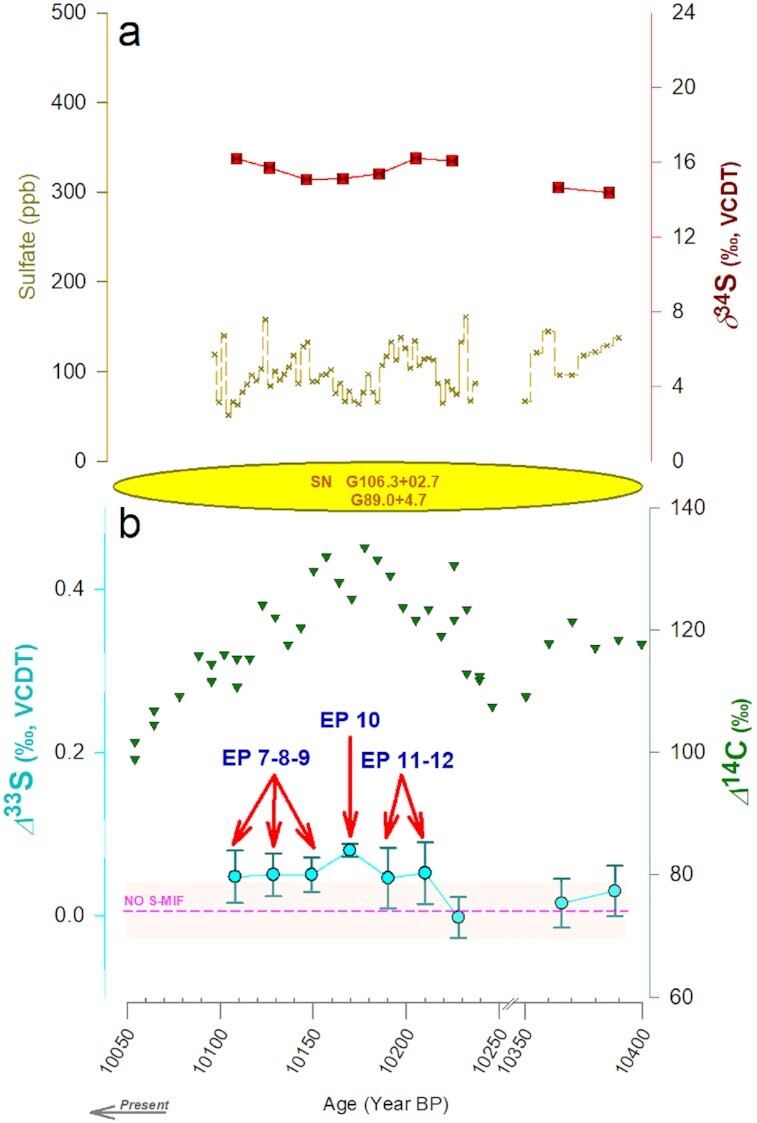
The evolution of ice-core sulfate isotopic signals during the multiple SN event. (a) Sulfate concentrations and δ^34^S values are reported (see more details in Table S2). The episodes marked with red arrows (“EPs”) highlight the case studies detailed in the discussion. The Δ^14^C record is obtained from CHRONO IntCal20 Database (qub.ac.uk). The SN remnants of similar ages are noted. The non-sea-salt sulfate fractions are shown in Figure S2. The AICC2012 chronology has been used (70, 71).

Ice-core sulfate concentrations and triple-S-isotope measurements were conducted with a high-resolution sampling of the Laschamp GE event (≈10.5 ± 2.5 y; Table S1) and the multiple SN events (≈20 ± 1.5 y; Table S2). Considerable variability was found in the sulfate concentrations and the corresponding isotopic signals during these events (Figures 2 and 3). For the GE event, the total sulfate concentrations, δ^34^S, and Δ^33^S values varied from 60 to 1200 ng/g, 5‰ to 15‰, and −0.1‰ to +0.5‰, respectively (Figure 2). The average sea-salt sulfate fraction was 13% ± 5% for this period (Figure S1). Likewise, for the SN event, the total sulfate concentrations, δ^34^S, and Δ^33^S values varied between 84 and 161 ng/g, 15‰ and 16‰, and −0.05‰ and +0.1‰, respectively (Figure 3). The estimated maximum contribution of sea-salt sulfate was <6% for this period (see the “Methods” section and Figures S1 and S2).

The S-isotopic characteristics are comparable during both GE and SN events (Figures 2 and 3). Several instances were detected wherein the sulfur-isotopic anomalies were present, i.e. Δ^33^S ≠ 0‰ and varying in magnitude across the span of the event. In principle, due to isotope mass-balance constraints (44), we would not have expected to see any such signals on a decadal/multi-decadal scale. However, here we have clear indications of primarily positive *Δ*^33^S during these events. It is likely that the negative S-isotopic anomalies created in the process are stored in other S-reservoirs (e.g. COS, SO, CS^2^) (38) and as such remain undetected in the present investigation. Some reports have also suggested detection of only positive anomalies in ice-core sulfate during volcanic eruptions and the negative anomalies were either lost or not detected in those particular ice cores (43–45). This remains a possibility in the present case as well. The δ^34^S values in both cases were remarkably stable throughout most of the event. It is confounding to witness dynamic shifts in the Δ^33^S values alone without any accompanying significant changes in the δ^34^S values. The relatively stable δ^34^S signal points to a consistent source fingerprint (47–50)—e.g. of sea-salt sulfate characterized by a δ^34^S value of 21‰ and of marine biogenic non-sea-salt sulfate with δ^34^S ranging from 12‰ to 19‰, whereas the dynamic Δ^33^S signals implicate a process/mechanism(s) (37, 38, 41, 47–51). Taken together, the evolution of the S-isotopic signals during the GE and SN events is intriguing and worth exploring in detail.

## Discussion

### Deciphering the origin of the observed *Δ*^33^S anomalies

Constantly trending Δ^33^S signals in ice-core sulfate were observed during both the Laschamp GE event (≈41 kBP) and the multiple SN event (≈10 kBP), respectively (Figures 2 and 3). The origin of these anomalies could be attributed to several different mechanisms. In general, photochemistry and atmospheric dynamics are involved in producing Δ^33^S anomalies in sulfate formed in the stratosphere (37, 38, 51), which are then detected in Antarctica, i.e. during stratospheric volcanic eruptions (44) (scenario b in Figure 1). S-MIF anomalies have only been detected during such “volcanic” periods in ice-core sulfate (41–46). This is because Antarctica's primary source of ice-core sulfate aerosols is marine biogenic emissions, also ubiquitous in polar snow (40–46). The sulfate formed from this source displays no significant S-MIF [*Δ*^33^S *Δ*0.05‰, similar to seawater; Table S3 in Gautier et al. (45) and Table 2 in Alexander et al. (40)]. Likewise, sulfate formed in the troposphere and found in the snow or soil of Antarctica has never shown any sulfur isotope anomaly either (40, 52) (Figure 1c). This is further evidenced in the year-round investigation of atmospheric sulfate aerosols in Antarctica, wherein the reported *Δ*^33^S was ≈0.01‰ on average for samples collected at both Dome C and Dumont D'Urville research stations (52). The volcanic sulfate (e.g. ∼41,250 BP in Figures 2a and S3) is superimposed on the marine-generated sulfate forming the continuous, low-concentration background (as seen in Figures 2a and 3a).

Other origins of S-MIF bearing ice-core sulfate may be possible (47–50, 53). Evidence of S-MIF has been found in aerosol samples in present-day polluted urban and semi-urban environments (47–50). In such locations, mineral-dust-associated sulfate has been proposed to exhibit S-MIF (47, 50), yet inherent mechanism(s) remain unclear and highly speculative. However, no such dust events have ever been reported for the period studied here (54), as also evidenced from the record of calcium concentrations (a marker for dust) in our dataset (Figure S1a; see the “Methods” section as well). Other than these, large-scale circulations such as strong El Niño Southern Oscillation (ENSO) have led to a positive *Δ*^33^S anomaly in Antarctic snow-pit samples (53). The positive values were then suggested to likely arise from carbonyl sulfide (COS) photochemistry following a strong stratosphere-troposphere exchange (53). However, this explanation is at odds with findings from laboratory experiments of COS photolysis wherein largely negative *Δ*^33^S anomalies were found in the process (49). Likewise, combustion experiments (associated with biomass burning and fossil fuel combustion) have also mainly shown negative *Δ*^33^S values (48, 49). They are less likely to explain the primarily positive *Δ*^33^S anomalies detected in the present investigation. The anthropogenic origin of nonzero *Δ*^33^S in modern-day sulfate aerosols (47–50) is, therefore, less likely to be relevant for ice-core sulfate found in the pristine Antarctic region. Taken together, photochemical processes—the better investigated, broadly understood, and widely accepted mechanism for nonzero *Δ*^33^S (positive and negative) values found in ice-core sulfate records (37, 38, 41–46)—could plausibly explain the isotopic anomalies observed during GE and SN events. To further deconvolute the dynamics of S-isotope anomalies during these periods, it is essential to scrutinize the “episodic” nature of the signals.

### Isotope forensics of the “episodes” (EPs)

**Fig. 4.**
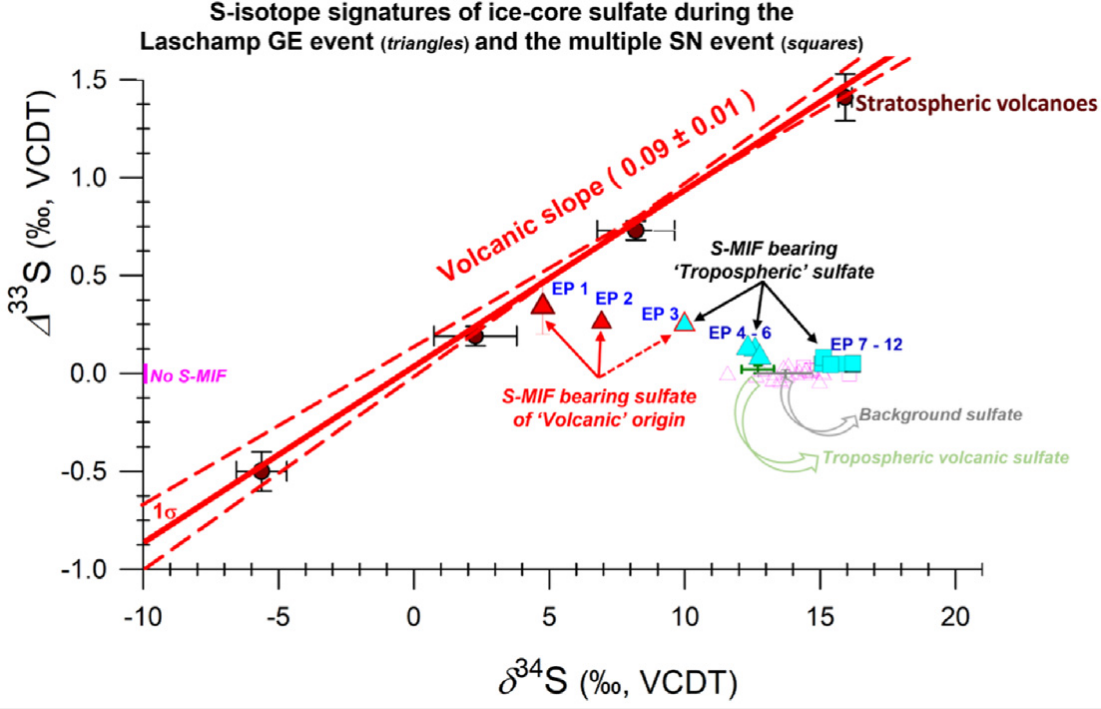
Triple-isotope-based deconvolution of the S-MIF signals during the GE and SN events. Five stratospheric volcanoes (circles), Kuwae, Samalas, 1809 UE, 1259 UE, and Agung, are shown. Also shown are all S-isotope values observed during the GE and SN events (in pink, bearing no S-MIF) and detected “Episodes (EPs)” (in cyan and red, bearing S-MIF) whose origin is shown with arrows (in red and black). The dotted arrow for EP 3 indicates only a peripheral “volcanic” component and a more significant contribution from the secondary tropospheric component for this isotope anomaly episode (see Discussion and Figure S3). The isotopic signatures of previously reported tropospheric volcanoes (45) and background sulfate (40–45) are shown. The isotopic values of the SN background sample measured in this study (Table S3) overlap with the previously reported background values.

The EPs—defined as nonzero *Δ*^33^S signals with analytical uncertainties not crossing the “no S-MIF” line (Figures 2 and 3)—both adhere to and deviate from the well-established mechanics of ice-core sulfate aerosols. In general, the sulfur isotope anomaly of the stratospheric sulfate follows a cyclic pattern—changing from positive Δ^33^S values at the beginning of the sulfate deposition to negative values at the end (41, 42, 44). The stratospheric SO_2_ oxidation kinetics leaves a consistent isotopic imprint on ice-core sulfate—the Δ^33^S vs δ^34^S slope is constrained to be 0.09 (*σ* = 0.02) (41–46) (Figure 4). This slope allows for distinguishing the “volcanic” vs. “nonvolcanic” event(s), which is crucial for deconvoluting the *Δ*^33^S dynamics.

Indeed, the Δ^33^S values on the higher extremes, as seen during both the GE and SN events (Figures 2 and 3), are comparable to those found during certain stratospheric volcanic events, e.g. the Tambora eruption (Δ^33^S = 0.15‰), Serua eruption (Δ^33^S = 0.16‰) detected in ice cores from Dome C, Antarctica (42–45). One such extreme in the observed Δ^33^S anomaly is EPs 1 to 3, which could be of volcanic origin owing to the high sulfate concentration, the closeness to the volcanic slope (Figure 4), and subsequent dynamics of the dual S-isotopes (Figure S3). Nonetheless, the decreasing 8^34^S signature of this episode, which is counter to the rising Δ^33^S values, is noteworthy (Figure S3) as it is contrary to the well-established SO_2_ oxidation kinetics as detailed above (44). Based on the progression of the subsequent signals, it can be argued that this is an extra-tropical volcano. In such a scenario, the volcanic plumes reach the sampling site faster than the counterpart carrying a stratospheric imprint (46), also causing a negative shift in the 8^34^S first (as volcanic sulfate has a 8^34^S ≈0‰ (55)).

The initial transport of an extra-tropical volcanic plume is expected to happen in the troposphere. It should ideally not be associated with a nonzero Δ^33^S when examined in ice-core sulfate records, as reported previously (46). As such, it can be argued that EP 3 could be associated with a “nonvolcanic” component displaying S-MIF. This is further supported by how the sampling of the volcanic event has been conducted (Figure S3). EP 3, sampled at the very beginning of the event, is composed mostly of samples reflecting background sulfate concentrations (see point “V_2_” in Figure S3). Together this indicates that EP 3 has peripheral influence from the volcanic component. Assuming our claim about EPs 1 and 2 being an extra-tropical volcano is correct, EP 3 implicates a secondary process possibly leading to S-MIF in tropospherically transported sulfate aerosols during this period. While EPs 1 and 2 potentially could explain the elevated Δ^33^S observed in a small section of the core, it does not fully explain the dynamics of the other “nonvolcanic” anomalies similar to EP 3 observed across the majority of the investigated ≈ 250 y of GE and SN event, respectively. The amplitude of isotopic shifts for nonvolcanic sulfate reported here has not yet been observed in any previous ice-core and aerosol sulfate-based S-MIF investigations in Antarctica (41–46, 52).

Evidence of tropospheric origin can be obtained further by investigating points with lower sulfate concentrations but higher Δ^33^S values. EPs 4 to 6 during the GE event are more attuned to be a background signal but exhibit an isotope anomaly, yet they do not fall on the volcanic slope (Figure 4). The sulfate concentrations and δ^34^S in EP 4 are comparable to that of the previously reported background values across different sites in Antarctica, such as at Dome C, the South pole (concentration of 80 ± 20 ng/g, δ^34^S of 14 ± 2‰) (43). However, the main difference is that no S-MIF has ever been reported in the background ice-core sulfate on several occasions (40–46). Remarkably, the source signature (likely of marine- biogenic origin) seems well preserved, while the S-MIF was observed in the ice-core sulfate. EPs 5 and 6 could still be tropospheric volcanoes. However, this does not seem to be the case compared to previously reported signals elsewhere (45) (Figure 4). We suggest that the S-isotopic anomalies, i.e. EPs 3, 4, 5, and 6 during the Laschamp GE event, were most likely of tropospheric origin.

Given the different kinds of events studied and the study's exploratory nature, a range of sampling resolutions was needed to understand the likelihood of detecting any peculiar signals. To further probe this aspect, we investigated another event with a potential for affecting the cosmic-ray background of Earth—the multiple SN event (Figure 3). EPs 7 to 12 during the SN event display background concentrations, yet all exhibit nonzero S-isotope anomalies. The magnitude of the EPs 7 to 12 is slightly muted relative to EPs 3 to 6, perhaps due to doubled sampling resolution for this event leading to further smoothening of the signal(s). A notable feature is a blip in the record, i.e. EP 10, which is significantly different (*P* < 0.05) than the rest of the EPs (7 to 12). The overlap of isotopic anomalies in these signals with EPs 4, 5, and 6, as seen in Figure 4, is further evidence of the notion that tropospheric sulfate aerosols were displaying S-MIF during the investigated period of the Laschamp GE event and the multiple SN event.

### A mechanism for generating S-MIF-bearing tropospheric sulfate during the GE and SN events

Based on the findings in this pilot study, we postulate an explanation for the observations in Figure 4. While the *Δ*^33^S dynamics contrast the well-established notion of SO_2_ oxidation kinetics imprinted in ice-core sulfate, they align with our ozone depletion hypothesis during the GE/SN events (scenario d in Figure 1).

Modeling studies have provided quantitative estimates for ozone depletion during such events (21, 24). For instance, during the Laschamp GE event, an estimated ∼5% ozone loss in the lower stratosphere over Antarctica with a similar increase in tropospheric ozone mixing ratios has been modeled (24). However, the observed geochemical signals contradict the modeled estimates. This is because more significant ozone losses (with even lower mixing ratios) occur in the short-term on an annual basis over present-day Antarctica. Nevertheless, no evidence of S-MIF in tropospheric sulfate has been found (52, 56, 57). Moreover, with most of the ozone layer intact in the lower stratosphere during the Laschamp GE event, increasing tropospheric ozone is likely to impede further S-MIF-generating UV-induced photochemical processes. This curtails detecting such signals in tropospheric sulfate in ice-core records altogether. Given the detection of such signals and on a decadal-scale resolution, we argue that a massive depletion/thinning of the ozone layer would have occurred. Therefore, these geochemical 'fingerprints are compelling to warrant further investigations on the estimated ozone loss during the Laschamp GE event.

Changes in the cosmogenic radionuclides accompanied the investigated periods. The period of the Laschamp GE event studied here was contemporaneous with elevated ^10^Be levels as documented in several studies (28–31). While ^10^Be was not measured for corresponding sections of the ice cores used in this study, it has been measured on Vostok 3 G cores in the past (28). The record is, however, discontinuous and of poor resolution (28). The same is available for Vostok 4 G cores but for a resolution of ∼100 y (58). ^10^Be measured on Vostok 5 G cores offered a similar time resolution (∼10 y as used in this study) (29). However, an ∼10 m offset in depth has been found between the 3 G and 5 G records (29). Taking this offset into account, we note that the ^10^Be concentrations were, on average, ∼290 ± 50.10^3^ atoms/g during this period (Figure S4). The corresponding ice-core 10Be measurements are unavailable for the multiple SN event. Therefore, we used another widely employed cosmogenic tracer *Δ*^14^ C. The measurements are from the global repository, the CHRONO IntCal20 Database, developed from various achieves (e.g. tree rings, sediments) ( 32). The resolution is similar to the ice core measurements conducted in this study (32). A blip is evident in the *Δ*^14^ C record during this period (Figure   3). Although other explanations are possible (59), this blip is also attributable to the multiple SN event (10). Notably, this is of global significance as the record is put together from achieves from various geographical regions (32). Here, we find a similar blip in the Antarctic ice-core Δ^33^S record—EP 10 to be concomitant with *Δ*^14^ C record (Figure   3).

Taken together, these fluctuations in the radionuclide and ice-core Δ^33^S records for the corresponding periods suggest that past GE/SN events had impacted the ozone layer simultaneously, creating a so-called UV “window” in the Earth's atmosphere. This led to the traceable enhanced UV-induced tropospheric photochemical imprints during these periods, thereby corroborating our hypothesis.

### Sporadic nature of the tropospheric photochemical imprints

Despite the likely existence of a UV “window” during the Laschamp GE event (≈41 kBP) and the multiple SN event (≈10 kBP), a continuous photochemical imprint (nonzero Δ^33^S values) was not witnessed in the Antarctic ice-core records. This can be attributed to different aspects, which could influence the detection of such signals:

1. The transport and dilution/mixing processes could play a significant role in this regard. The magnitude of S-MIF detected in ice-core sulfate would be governed by sulfate formed from competing processes (photolysis vs. photoexcitation) (37, 38, 44, 51). However, once formed, these tropospheric aerosols might get deposited near the surface and not make it to the poles because of the prevailing meteorology. They might also get diluted en route with sulfate formed from the dominant nonphotochemical pathway (SO_2_ + OH reaction), which is not associated with any isotopic anomaly (60).
2. The role of site-specific characteristics, e.g. the regional pattern of snow accumulation, a central variable when investigating the evolution of polar ice sheets and their sensitivities to changes in the atmosphere/climate, needs to be considered. We speculate that the difference in accumulation rates is likely to affect the frequency of detection of the episodic events between different ice-core records from Northern Hemispherical polar sites (e.g. in Greenland) having a higher snow accumulation rate [nearly an order higher in some regions (61)] vs. most of the Antarctic sites, e.g. Vostok used for this study (62, 63). A multisite investigation would be needed to corroborate this aspect and remains beyond the scope of this study.
3. The influence of field morphology and the potency of the UV “window” are critical factors. In the ∼2000 y of the Laschamp GE, the Earth's magnetic field (and the relative paleomagnetic intensity) featured several changes (24, 64) that can be grouped as the period of initial collapse (∼−42.3 to −41.6 kBP), the period of partial recovery (−41.5 to −41.2 kBP), the period of partial collapse (−41.1 to −40.9 kBP), the period of final recovery (−40.8 to −40 kBP). During these periods, the modeled evolution of the paleomagnetic intensities exhibits latitudinal and hemispherical differences (64). As such, the formation of the UV “window” (in terms of its hemispherical location) is expected to vary concomitantly to the changes in field morphology. The S-MIF signals would also vary in magnitude and frequency between, e.g. Greenland vs. Antarctic ice-core records. The scale and potency of the UV window are essential aspects to consider. The present study investigated such signals during partial recovery of the magnetic field. As such, the UV “window” is expected to have slightly recovered as well relatively. This would consequently lead to changes in the magnitude of S-MIF signatures of the tropospheric sulfate.

To further test our findings, we conducted a separate analysis to gain insight into the background Δ^33^S values in the period before the SN event (≈11.2 kBP). This analysis did not reveal any S-MIF anomaly (Table S3). Likewise, a separate analysis was also conducted for the GE event ≈42.3 kBP. This period coincides with the onset of changes in paleomagnetic intensities and cosmogenic radionuclide records (24). However, we did not find any S-MIF anomalies either. This suggests that the trending signals are indeed acquired during the events.

The observational evidence presented here shows a new application for ice-core-Δ^33^S records—as a potential proxy for ozone layer depletion events. This pilot study can thus initiate the investigation of similar events to improve our understanding of the interaction between cosmic events and associated environmental stress for past and future events.

## Materials and methods

Ice cores were drilled at the Vostok (VK-3 G) site in Antarctica, as detailed elsewhere (54). High-resolution sampling was conducted by cutting ∼3 cm slices along the wings of the cores. Outside edges of the ice core were scraped clean, and ice-core slices were then decontaminated with Milli Q water, following which the samples were melted. The concentration measurements were made using an ion chromatography Metrohm IC (Professional 850) for each slice requiring 2 ml of the sample. Given that large variability in various chemical parameters has been reported during the Laschamp GE period (≈ 41 kBP) (54), e.g. in the sodium concentrations, major water-soluble ions were measured in the samples covering 300 y of the Laschamp event studied here (Figure S1, Figure 2). As no such variability was seen in the period covering the SN event (≈10 kBP), and the sodium and dust concentrations were also an order lower compared to the Laschamp period (54), only sulfate concentrations were measured in the samples covering the SN event (Figure 3).

Following the IC analysis, the sulfate from pooled samples was isolated using IC-based separation at Institut des Géosciences de l'Environnement (IGE) (45) (special care was taken not to merge samples with peak concentrations and samples with background-level concentrations). The extracts were then analyzed for S-isotopes at CRPG with the Neptune Plus MC-ICP-MS (Thermo Fisher Scientific Neptune Plus) coupled with a desolvation membrane (Aridus II, Cetac) to reduce the interference of oxides and hydrides on the masses ^32^S, ^33^S, and ^34^S and to increase the sensitivity to the signal (65–68). The samples are analyzed by standard-sample bracketing using an in-house Na_2_SO_4_ standard to correct for instrumental isotopic fractionation (65). Before introducing the sample to the instrument, the sulfate extracts were evaporated to dryness, resuspended in 5% HNO_3_, and NaOH was added to the sample to match the concentration and matrix of the in-house Na_2_SO_4_ bracketing standard with a concentration of 40 μmol/L. As part of quality control, the bracketing standard was measured as a sample with decreasing sulfate concentration, showing no bias induced by sulfate concentration as long as Na concentration matches that of the bracketing standard (Table S4), in agreement with Paris et al.(65).

Each sample was run a minimum of four independent times, usually five, with each run making fifty measurements and a sulfate concentration between 20 and 40 μmol/L. The reported values (see Tables S1 and S2) are given as an average of at least five independent measurements on the Neptune for a given sample. This analytical error (usually 0.05 ‰ to 0.1 ‰, 2σ) is an improvement from previous MC-ICPMS-based studies (65–68) as samples are run (i) at a higher concentration (up to 40 μmol/l instead of less than 20 μmol/L) and (ii) five times instead of two, respectively. The error accounts for instrumental reproducibility. The sample required for this method is ≈40 nmol, and as such, this could become a preferable method for high-resolution analysis of precious ice core archives.

The triple sulfur isotopes (^32^S, ^33^S, ^34^S) are reported relative to Vienna-Canyon Diablo Troilite (V-CDT). The bracketing standard was calibrated against the international standard IAEA-S1 assuming δ^34^S = −0.3‰ and δ^33^S = −0.061‰ (and thus Δ^33^S = 0.094‰). Further quality control was conducted using a secondary sulfur isotope reference material “SMIF-2” which yielded a δ^34^S value of 10.06‰ ± 0.14‰ and Δ^33^S of 9.46‰ ± 0.14‰ (*n* = 5) in agreement with the reported Δ^33^S = 9.54‰ ± 0.09‰ from five different laboratories worldwide (69). A seawater sample was also run further to ensure the precision and accuracy of the measurements. It yielded δ^34^S and Δ^33^S values of 21.14‰ ± 0.09‰ and 0.07‰ ± 0.12‰ (2 sd, *n* = 5), in agreement with previously published values (65–68). The seawater sample was purified at CRPG following a column chemistry method (65, 66). Procedural blanks were also run with each set of samples. Overall, high-precision measurements of both δ^34^S and Δ^33^S were ensured during this investigation.

## Supplementary Material

pgac170_Supplemental_File

## Data Availability

All data are included in the manuscript and/or supporting information.
