# Supplementary material for: Sulfur-isotope anomalies recorded in Antarctic ice cores as a potential proxy for tracing past ozone layer depletion events
Source: PNAS Nexus. 2022 Aug 30;1(4):pgac170. doi: 10.1093/pnasnexus/pgac170 (PMC9802080; doi:10.1093/pnasnexus/pgac170)
Supplement: pgac170_Supplemental_File [file pgac170_supplemental_file.pdf]

Supplementary Information for

Sulfur-isotope anomalies recorded in Antarctic ice cores as a potential proxy for tracing past ozone layer depletion events

Sanjeev Dasari[1], Guillaume Paris[2], Julien Charreau[2], Joel Savarino[1*]*

[1]Institut des Géosciences de l'Environnement (IGE), University Grenoble Alpes, CNRS, IRD, Grenoble INP, Grenoble 38000, France

[2]Université de Lorraine, CNRS, CRPG, 54000 Nancy, France

To whom correspondence should be addressed

Email: sanjeev.dasari@univ-grenoble-alpes.fr

joel.savarino@univ-grenoble-alpes.fr

Supporting information includes: 12 pages, 4 Figures, 4 Tables

Contents

Supplementary Figures

Figure S1. Concentrations of water-soluble ions and source fractions of sulfate …………………………...S3
Figure S2. Non-sea-salt sulfate concentrations during the Laschamp GE event (≈ 41 kBP) and the multiple SN event (≈ 10 kBP)…………..……..……………………………………..…………………………………S4
Figure S3. Sampling of the volcanic event ………………………………………………………………S5
Figure S4. ^{10}Be concentrations in Vostok 5G cores ……………………………………………………...S6

Supplementary Tables

Table S1. S-isotope values for samples analyzed during the Laschamp GE event. …………………………S7
Table S2. S-isotope values for samples analyzed during the multiple SN event……......................................S9
Table S3. S- isotope values for samples analyzed before the multiple SN event and onset of the Laschmap GE event….…………………………………………………….. …………………………………S10
Table S4 Measurement accuracy with decreasing sulfate concentration.…..…………………...…………S11

References

Supplementary Figures

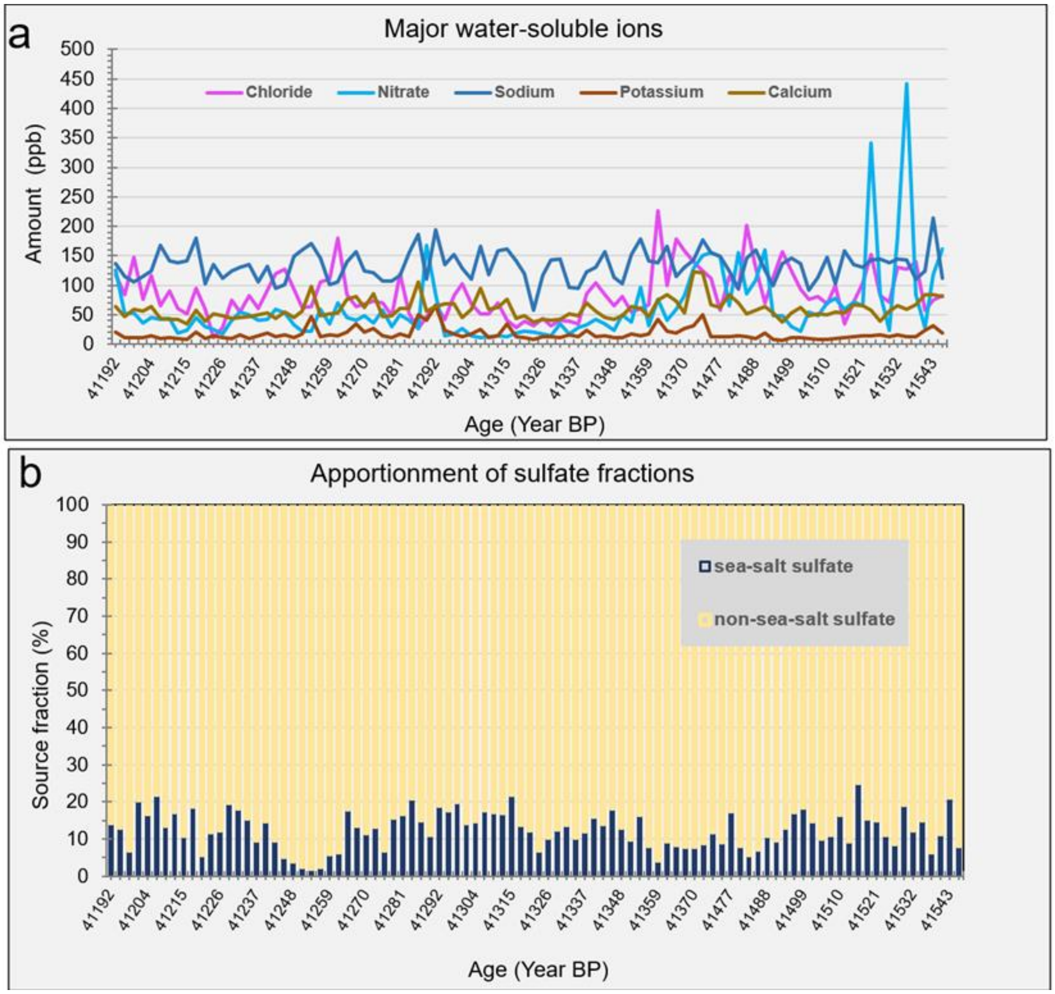

Figure S1. Concentrations of water-soluble ions and source fractions of sulfate. (a) Major ions measured for the investigated period of the Laschamp Geomagnetic excursion event are shown. (b) The sulfate source fractions are obtained as a ratio of the non-sea-salt sulfate and the sea-salt sulfate concentrations to the total sulfate concentrations, respectively. The non-sea-salt sulfate concentrations are, in general, calculated by subtracting total sulfate concentrations (data shown in Figures 2 and 3 in the main manuscript) and the product of sodium concentrations and the [SO_4^{2-}]/[Na^+] mass ratio (represented as *k*) in sea salt particles[1]. A *k* value in seawater of 0.25 is often used for this calculation[2,] but in Antarctica, due to temperature differences, the sea salt emitted from the sea ice surface is depleted in SO_4^{2-} relative to Na^+ [3]. As such, lower values have been reported at various Antarctic sites[4,5], e.g., at Dome C (0.16 ± 0.09) and DDU (0.13 ± 0.04), respectively. Therefore, here the average *k* value of 0.14 has been used.

For the 300 years of the Laschamp period investigated in this study, the average sea-salt sulfate fraction is 13±5 % [using the *k* value of 0.25, this fraction was found to be 20±9 %]. For the Supernova event (≈ 10 kBP), based on the reported maximum sodium concentration for the corresponding depths/ages[6], we have estimated the maximum fraction of sea-salt sulfate for this period to be < 6 % [using the *k* value of 0.25, this fraction was found to be < 9 %]. The non-sea-salt sulfate concentrations for both these events are shown here in Figure S2.

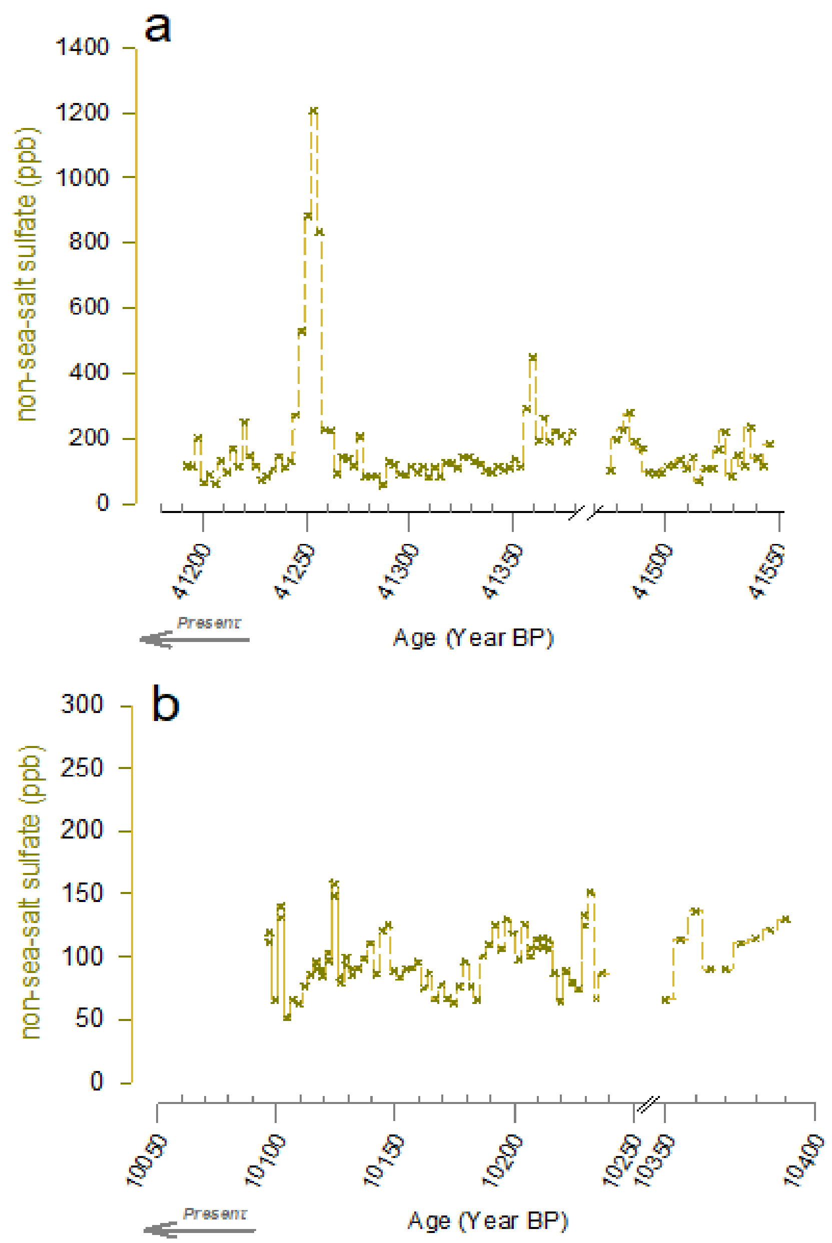

Figure S2. Non-sea-salt sulfate concentrations during the Laschamp GE event (≈ 41 kBP) and the multiple SN event (≈ 10 kBP). See details of sulfate source apportionment in Figure S1.

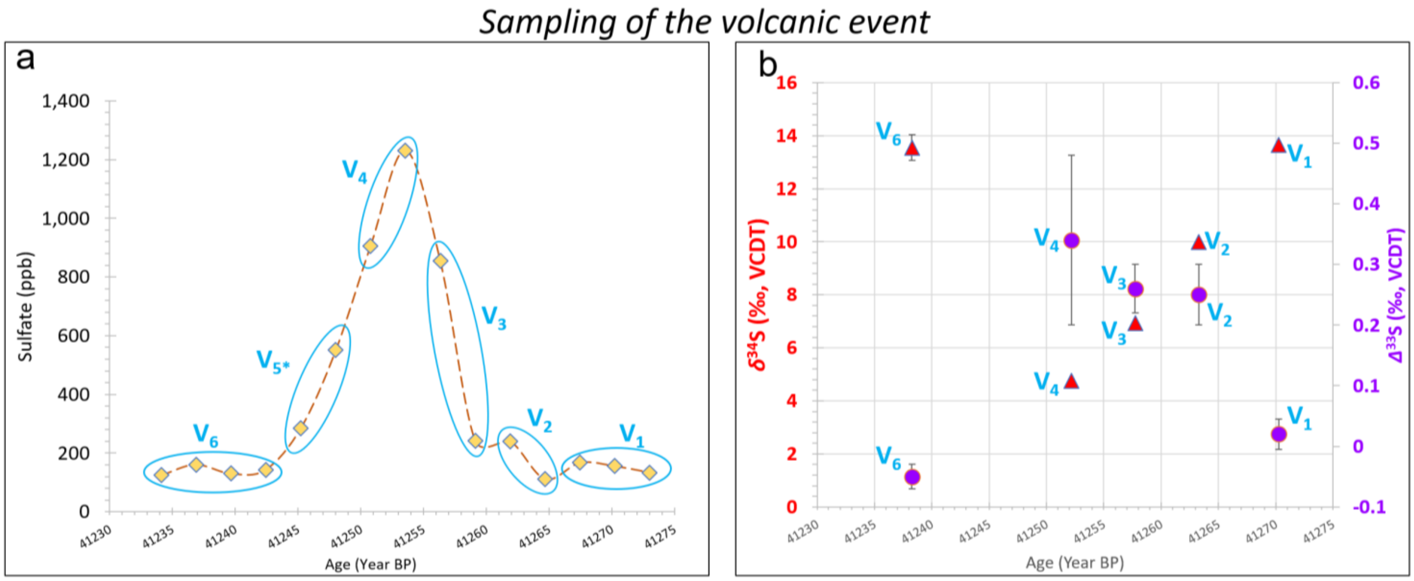

Figure S3. Sampling of the volcanic event. The pooled samples are here shown with the circles and prefix 'V' followed by the sample number (a). The corresponding S-isotope compositions are also shown (Δ^{33}S in 'purple circles' and δ^{34}S in 'red triangles') (b). Sample 5 (V_5, marked with *) was lost during the evaporation process as no sulfate was detected in the MC-ICP-MS run. The error bar on sample V_4 is larger than the rest of the samples as only two successful replicate runs were possible for this sample due to issues with instrument stability during other runs, which were discarded.

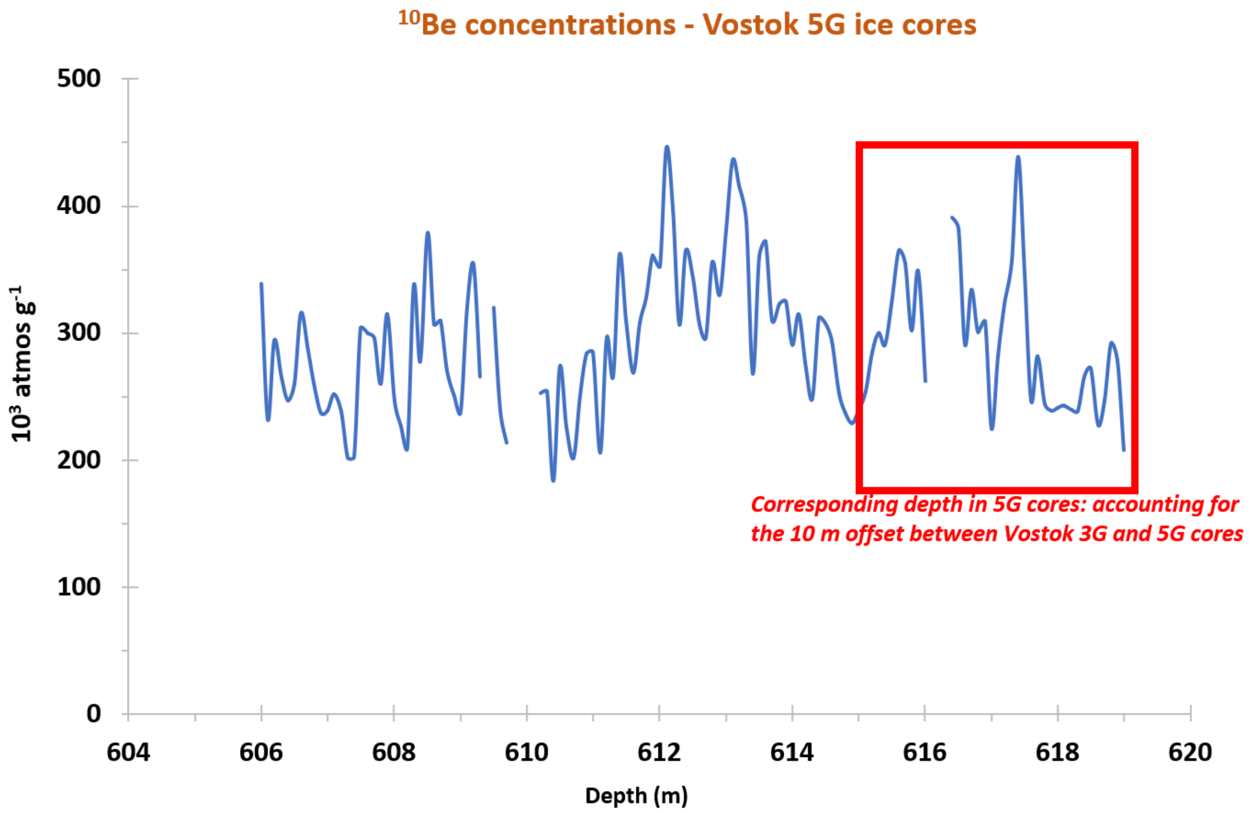

Figure S4. ^{10}Be concentrations in Vostok 5G cores. The highlighted depth range corresponds to the depths of Vostok 3G cores used in this study for the S-isotope measurements and accounts for the ~10 m offset between Vostok 3G and 5G cores[7].

Supplementary Tables

Table S1. S-isotope values for samples analyzed during the Laschamp GE event. The mean and standard deviations of the ensemble of the measurements are shown. Sample 5 was lost during the evaporation process as no sulfate was detected in the MC-ICP-MS run. Only two successful replicate runs were possible for Sample 6 due to issues with instrument stability during other runs, which were discarded.

Sample ID	Average Depth (m)	Average Age (year BP)	δ^{34}S(‰)		Δ^{33}S(‰)	
			Mean	s.d.	Mean	s.d.
1	606.09±0.05	41199±4	13.79	0.12	-0.02	0.04
2	606.24±0.05	41212±4	14.73	0.14	0.01	0.05
3	606.39±0.05	41226±4	11.58	0.08	-0.01	0.05
4	606.52±0.04	41238±4	13.55	0.03	-0.05	0.02
5*	606.61±0.02	41247±2	NA	NA	NA	NA
6	606.67±0.02	41252±2	4.76	0.02	0.34	0.14
7	606.73±0.02	41258±2	6.93	0.48	0.26	0.04
8	606.79±0.02	41263±2	9.98	0.04	0.25	0.05
9	606.87±0.03	41270±3	13.65	0.06	0.02	0.03
10	606.96±0.03	41281±3	12.84	0.04	0.01	0.05
11	607.05±0.03	41291±3	14.44	0.02	-0.01	0.03
12	607.15±0.04	41302±4	14.46	0.02	0.01	0.02
13	607.27±0.04	41311±4	14.5	0.02	0.01	0.02
14	607.39±0.04	41321±4	12.64	0.08	-0.02	0.04
15	607.51±0.04	41331±4	12.6	0.08	0.12	0.05
16	607.63±0.04	41341±4	14.98	0.04	-0.05	0.02
17	607.74±0.03	41351±3	13.31	0.04	-0.01	0.04
18	607.83±0.03	41359±3	12.33	0.12	0.13	0.02
19	607.90±0.03	41368±3	14.22	0.09	0	0.03
20	607.98±0.02	41379±3	13.51	0.15	-0.04	0.04
21	609.07±0.04	41480±4	12.78	0.11	0.08	0.03
22	609.19±0.04	41492±4	15.13	0.03	-0.01	0.02
23	609.31±0.04	41503±4	15.08	0.03	0	0.04

24	609.43±0.04	41514±4	13.83	0.03	0.03	0.03
25	609.54±0.04	41523±3	13.82	0.09	-0.01	0.06
26	609.63±0.04	41532±3	13.26	0.06	-0.04	0.04
27	609.73±0.04	41541±4	14.13	0.12	-0.01	0.05

Table S2. S-isotope values for samples analyzed during the multiple SN event. The mean and standard deviations of the ensemble of the measurements are shown.

Sample ID	Average Depth (m)	Average Age (year BP)	$\delta^{34}S$(‰)		$\Delta^{33}S$(‰)	
			Mean	s.d.	Mean	s.d.
1	236.25±0.14	10107±7	16.20	0.01	0.05	0.03
2	236.68±0.12	10128±6	15.72	0.03	0.05	0.03
3	237.08±0.12	10148±6	15.08	0.04	0.05	0.02
4	237.48±0.12	10168±6	15.13	0.05	0.08	0.01
5	237.87±0.12	10188±6	15.39	0.11	0.05	0.04
6	238.27±0.12	10208±6	16.22	0.05	0.05	0.04
7	238.67±0.12	10228±6	16.08	0.12	0.00	0.03
8	241.35±0.19	10362±9	14.65	0.07	0.02	0.03
9	241.80±0.10	10385±5	14.39	0.07	0.03	0.03

Table S3. S- isotope values for samples analyzed before the multiple SN event and onset of the Laschmap GE event. These samples were analyzed for the period before the SN event (≈11.2 kBP) and at the onset of the GE event (≈42.3 kBP), respectively, with no history of volcanic activity in this core and sulfate concentration 120±10 ng/g.

**Sample no.**	**$\delta^{34}S$(‰)**	**$\Delta^{33}S$(‰)**
**SN event**	14.33	0.00
	14.33	0.00
	14.38	0.00
**GE event**	11.93	0.02
	11.90	0.02
	11.88	0.00

Table S4. Measurement accuracy with decreasing sulfate concentration (all samples with the same Na concentration as the bracketing standard 40 µmol/L Na_2SO4)

umol/L	$\delta^{34}S$(‰)	2sd	$\Delta^{33}S$(‰)	2sd
40	4.43	0.09	0.02	0.07
30	4.31	0.14	0.00	0.10
20	4.52	0.15	-0.01	0.12
calibrated value	4.47	0.05	0.00	0.05

References

1. Ishino S, Hattori S, Savarino J, Legrand M, Albalat E, Albarede F, Preunkert S, Jourdain B, Yoshid N. 2019. Homogeneous sulfur isotope signature in East Antarctica and implication for sulfur source shifts through the last glacial-interglacial cycle. Sci Rep. 9:1-8.

2. Holland H D, Lazar B, Mccaffrey M. 1986. Evolution of the Atmosphere and Oceans. Nature. 320:27–33.

3. Wagenbach D, et al. 1998. Sea-salt aerosol in coastal Antarctic regions. J Geophys Res Atmos. 103:10961–10974.

4. Legrand M, et al. 2017. Year-round records of bulk and size-segregated aerosol composition in central Antarctica (Concordia site) – Part 1: Fractionation of sea-salt particles. Atmos Chem Phys. 17:14039–14054.

5. Jourdain B, Legrand M. 2002. Year-round records of bulk and size-segregated aerosol composition and HCl and HNO3 levels in the Dumont d'Urville (coastal Antarctica) atmosphere: Implications for sea-salt aerosol fractionation in the winter and summer. J Geophys Res Atmos. 107:4645.

6. Petit J R, Jouzel J, Raynaud D, Barkov N I, Barnola J M, Basile I, Bender M, Chappellaz J, Davis M, Delaygue G, Delmotte M. 1999. Climate and atmospheric history of the past 420,000 years from the Vostok ice core, Antarctica. Nature. 399:429-436.

7. Raisbeck G M, Cauquoin A, Jouzel J, Landais A, Petit J R, Lipenkov V Y, Yiou F. 2017. An improved north–south synchronization of ice core records around the 41 kyr 10 Be peak. Climate of the Past. 13:217-229.
